# Aufmerksamkeitsdefizit‑/Hyperaktivitätsstörung und kardiometabolische Erkrankungen: Prävalenz, Ätiologie und Behandlung

**DOI:** 10.1007/s00115-025-01828-4

**Published:** 2025-04-29

**Authors:** Sarah Kittel-Schneider

**Affiliations:** 1 Department of Psychiatry and Neurobehavioural Science, Acute Adult Mental Health Unit, Cork University Hospital, University College Cork, T12DC4A Wilton, Cork, Irland; 2 APC Microbiome, University College of Cork, Cork, Irland; 3 Klinik für Psychiatrie, Psychotherapie und Psychosomatik, Universitätsklinikum Würzburg, Würzburg, Deutschland

**Keywords:** Metabolische Erkrankungen, Adipositas, Kardiovaskuläre Erkrankungen, Genetik, Lebensstil, Metabolic diseases, Obesity, Cardiovascular diseases, Genetics, Lifestyle

## Abstract

Zwischen der Aufmerksamkeitsdefizit‑/Hyperaktivitätsstörung (ADHS) und einem erhöhten Risiko für kardiometabolische Erkrankungen wie Adipositas, Diabetes mellitus und arterielle Hypertonie besteht eine enge Verbindung über die gesamte Lebensspanne. Ursächlich sind nach heutigem Wissen genetische Faktoren, Dopaminstoffwechselstörungen, zirkadiane Rhythmusstörungen, Entzündungsprozesse und nicht zuletzt ungesunder Lebensstil. Die Behandlung von Individuen mit ADHS und komorbiden kardiometabolischen Erkrankungen erfordert einen individualisierten Ansatz mit Lebensstiländerungen, Psychotherapie und medikamentöser Therapie unter Berücksichtigung möglicher kardiometabolischer Nebenwirkungen der Medikamente.

## Abstract (en)

There is a close connection throughout the lifespan between attention deficit/hyperactivity disorder (ADHD) and an increased risk of cardiometabolic diseases, such as obesity, diabetes mellitus and arterial hypertension. According to current knowledge, the causes are genetic factors, dopamine metabolism disorders, circadian rhythm disorders, inflammatory processes and, last but not least, an unhealthy lifestyle. The treatment of individuals with ADHD and comorbid cardiometabolic diseases requires an individualized approach with lifestyle changes, psychotherapy and pharmacotherapy, taking possible cardiometabolic side effects of the ADHD medication into account.

## Lernziele

Nach Lektüre dieses Beitrags …

- können Sie die häufigsten komorbid auftretenden kardiometabolischen Erkrankungen bei der Aufmerksamkeitsdefizit‑/Hyperaktivitätsstörung (ADHS) benennen,
- sind Sie in der Lage, die zugrunde liegenden Zusammenhänge zwischen ADHS und kardiometabolischen Erkrankungen abzuleiten
- können Sie sicher die passende psychopharmakologische Medikation auch bei komorbiden metabolischen Erkrankungen bei ADHS verwenden,
- kennen Sie die passende multimodale Behandlung bei ADHS-Patienten mit kardiometabolischen Erkrankungen.

## Hintergrund

Die Aufmerksamkeitsdefizit‑/Hyperaktivitätsstörung (ADHS) ist die häufigste **entwicklungspsychiatrische Erkrankung**

entwicklungspsychiatrische Erkrankung,

die sich durch Unaufmerksamkeit, Hyperaktivität und Impulsivität auszeichnet. Es ist schon lange bekannt, dass ADHS-Betroffene ein deutlich erhöhtes Risiko für die Entwicklung **komorbider psychischer Erkrankungen**

komorbider psychischer Erkrankungen

haben, wie z. B. affektive Störungen, Angststörungen und Suchterkrankungen. Es gibt aber auch zunehmend Evidenz für eine enge Beziehung zwischen ADHS und einer Reihe **somatischer Komorbiditäten**

somatischer Komorbiditäten,

insbesondere kardiometabolische Erkrankungen. Daher ist es auch für die psychiatrische Behandlung bei ADHS-Betroffenen wichtig, diese Risiken zu kennen und in einer individualisierten Therapie mit zu beachten.

### Fallbeispiel

Herr M., 32 Jahre alt, stellte sich mit einem BMI von 42 kg/m^2^ und einem schlecht eingestellten Diabetes mellitus Typ 2 (HbA1c 9,2 %) in der endokrinologischen Ambulanz vor. Trotz mehrfacher Versuche der Lebensstilmodifikation und oraler Antidiabetika gelang es ihm nicht, sein Gewicht zu reduzieren oder seinen Blutzucker zu kontrollieren.

Bei der Anamnese fielen Konzentrationsschwierigkeiten und Impulsivität auf, Impulsivität insbesondere beim Essverhalten. Eine psychiatrische Evaluation führte zur Diagnose einer adulten ADHS. Nach Beginn einer Behandlung mit Methylphenidat (30 mg/Tag) berichtete Herr M. von einer verbesserten Impulskontrolle und Konzentrationsfähigkeit.

Innerhalb von 6 Monaten verlor Herr M. 15 kg Körpergewicht (BMI-Reduktion auf 37 kg/m^2^). Er gab an, nun besser Mahlzeiten planen und Heißhungerattacken widerstehen zu können. Parallel dazu verbesserte sich seine Blutzuckereinstellung deutlich, mit einem Abfall des Hämoglobin-A1c(HbA1c)-Wertes auf 6,8 %.

Die Kombination aus ADHS-Behandlung und verbesserter Selbststeuerung ermöglichte Herrn M. eine effektivere Umsetzung der Lebensstilempfehlungen. Dieser Fall unterstreicht die Bedeutung der Erkennung und Behandlung von ADHS bei Patienten mit Adipositas und Diabetes mellitus Typ 2, da dies zu einer signifikanten Verbesserung des Gesamtgesundheitszustands führen kann.

## Prävalenz

Mehrere epidemiologische Studien haben gezeigt, dass ADHS-Patienten ein erhöhtes Risiko für **metabolische Störungen**

metabolische Störungen

haben und umgekehrt. Metabolische Störungen umfassen eine Vielzahl von Erkrankungen, die den Stoffwechsel des Körpers beeinflussen, wie z. B. Adipositas und Diabetes mellitus (DM). Diese Kombination von Erkrankungen erhöht das Risiko für Herz-Kreislauf-Erkrankungen, zerebrale Ischämien und andere gesundheitliche Komplikationen erheblich. Zu den metabolischen Erkrankungen gehören mehrere Störungen, die oft gemeinsam auftreten und als **metabolisches Syndrom**

metabolisches Syndrom

bezeichnet werden. Die Hauptkomponenten sind:

1.  Adipositas, insbesondere mit Fetteinlagerung im Bauchbereich,
2.  erhöhter Blutzuckerspiegel oder Typ-2-Diabetes,
3. arterielle Hypertonie und
4. Dyslipoproteinämie, gekennzeichnet durch erhöhte Triglyzeride und/oder niedriges HDL-Cholesterin [1].

Im Folgenden werden wir die Prävalenzen der einzelnen metabolischen Erkrankungen bzw. Komponenten des metabolischen Syndroms und assoziierter kardiovaskulärer Erkrankungen im Zusammenhang mit ADHS genauer beschreiben.

### Adipositas

Übergewicht ist durch einen **Body-Mass-Index**

Body-Mass-Index

(BMI) von ≥ 25 kg/m^2^ bei Erwachsenen und bei Kindern durch eine **BMI-Perzentile**

BMI-Perzentile

> 90–97 definiert. Adipositas ist durch einen BMI von > 30 kg/m^2^ bei Erwachsenen und bei Kindern durch eine BMI-Perzentile > 97–99,5 definiert [2]. Adipositas ist eine der am häufigsten berichteten Komorbiditäten bei ADHS. Metaanalysen haben eine signifikante Assoziation zwischen ADHS und Adipositas gefunden, wobei die gepoolte Prävalenz von Adipositas bei ADHS-Patienten um etwa 40 % bei Kindern und Jugendlichen und 70 % bei Erwachsenen erhöht ist [3]. Anders gesagt, ergab eine rezente Metaanalyse, welche 1,4 Mio. Personen einschloss, eine Odds Ratio (OR) von 1,32 (95 %-Konfidenzintervall[KI] 1,18–1,47) für Adipositas bei Kindern und Jugendlichen mit ADHS [4]. Für Erwachsene wurde z. B. in einer schwedischen Registerstudie mit 2,5 Mio. Individuen eine **Risk Ratio**

Risk Ratio

von 3,05 (95 %-KI 2,95–3,15) gefunden [5].

### Diabetes mellitus

Mehrere Studien haben gezeigt, dass ADHS-Patienten auch ein erhöhtes Risiko für Diabetes mellitus (DM) Typ II haben, was unter anderem eine **Folgeerkrankung**

Folgeerkrankung

einer Adipositas sein kann [6]. Eine Längsschnittstudie ergab, dass ADHS-Patienten mit einer Hazard Ratio (HR) von 2,84 mit größerer Wahrscheinlichkeit DM Typ II im Erwachsenenalter entwickeln als Kontrollpersonen. Frühere Studien deuten auch auf eine Assoziation zwischen mütterlichem **Schwangerschaftsdiabetes**

Schwangerschaftsdiabetes

und einem erhöhten ADHS-Risiko bei den Nachkommen hin [6], allerdings zeigte eine neuere Metaanalyse zwar ein erhöhtes Risiko für Autismusspektrumstörungen (ASD) bei den Nachkommen (OR 1,42; 95 %-KI 1,22–1,65), aber nicht für ADHS (OR 1,01; 95 %-KI 0,79–1,28) [7].

### Arterielle Hypertonie und kardiovaskuläre Erkrankungen

Erwachsene mit ADHS scheinen ein erhöhtes Risiko für kardiovaskuläre Erkrankungen, einschließlich arterieller Hypertonie zu haben. In einer **Metaanalyse**

Metaanalyse

von Larsson et al. von 2023 wurden insgesamt wurden 18 Mio. Personen aus 11 Studien in eine **systematische Übersichtsarbeit**

systematische Übersichtsarbeit

einbezogen und 8 Mio. Personen aus 5 Studien in der Hauptmetaanalyse zu dem erhöhten Risiko von kardiovaskulären Erkrankungen analysiert. Die gepoolten Schätzungen zeigten, dass ADHS signifikant mit einem erhöhten Risiko für kardiovaskuläre Erkrankungen verbunden war (OR = 1,96; 95 %-KI = 1,19–2,23). In 5 der eingeschlossenen Studien wurde speziell das Risiko für arterielle Hypertension untersucht, in den anderen Studien wurden alle Arten von kardiovaskulären Erkrankungen zusammengefasst wie z. B. Myokardinfarkt, Schlaganfall, Vorhofflimmern, Herzinsuffizienz. In Subgruppenanalysen fanden die Autoren ein erhöhtes Risiko für Herz-Kreislauf-Erkrankungen in Verbindung mit ADHS über alle Altersgruppen, Arten von Herz-Kreislauf-Erkrankungen und Datenquellen hinweg [8].

### Dyslipidämien

Die Datenlage zu Fettstoffwechselstörungen bei ADHS ist noch recht spärlich und eher inkonsistent. In einer aktuellen deutschen Studie wurde **keine signifikante Assoziation**

keine signifikante Assoziation

(bei Frauen HR: 1,04; 95 %-KI: 0,84–1,28 und bei Männern HR: 0,89; 95 %-KI: 0,74–1,06) zwischen ADHS und Stoffwechselstörungen in einer retrospektiven Kohorte von 5367 Patienten mit ADHS aus den Datenbanken von Hausarztpraxen und 26.835 gematchten Kontrollen gefunden [9]. In einer longitudinalen Nachverfolgung von Kindern und Jugendlichen mit ADHS-Symptomen bzw. ADHS-Diagnose (ca. 240 aus einer Gesamtstichprobe von ca. 8000 Personen) konnte erneut ein höherer BMI (um im Schnitt *B* = 0,92 kg/m^2^), ein höherer systolischer (im Schnitt 3,5 mm Hg) und diastolischer (im Schnitt 2,2 mm Hg) Blutdruck gefunden werden. Und hier gab es auch Hinweise auf höhere Triglyzeridwerte (im Schnitt 0,24 mmol/l), und mehr Personen mit einer ADHS in der Kindheit waren Raucher (OR = 1,6). Ein statistisch signifikanter Zusammenhang mit LDL-Cholesterin wurde hingegen nicht gefunden [10].

#### Merke

Die diagnostische Abklärung kardiometabolischer Erkrankungen ist wichtig bei Patienten mit ADHS, weil diese ein höheres Risiko als die Allgemeinbevölkerung haben.

## Pathomechanismen

Die Mechanismen, die den Zusammenhang zwischen ADHS und metabolischen Störungen erklären, sind komplex und multifaktoriell. Untere anderem könnten hier ein **niedrigerer Bildungsstand**

niedrigerer Bildungsstand,

ungünstige Lebensstilfaktoren und psychiatrische Komorbiditäten erklärend sein. Außerdem gibt es gemeinsame **genetische Faktoren**

genetische Faktoren,

die eine Rolle spielen könnten. Mehrere potenzielle Pathomechanismen wurden vorgeschlagen, auf die hier kurz eingegangen werden soll:

### Gemeinsame Risikogene

Genetische Studien haben gezeigt, dass ADHS und metabolische Störungen einige gemeinsame genetische Risikovarianten teilen. Genomweite Assoziationsstudien (GWAS) haben signifikante genetische Korrelationen zwischen ADHS und Body-Mass-Index (**BMI**

BMI)

sowie zwischen ADHS und **Diabetes mellitus Typ 2**

Diabetes mellitus Typ 2

identifiziert (für ein Review siehe [6]). Auch gibt es Hinweise auf einen genetischen Zusammenhang zwischen ADHS und **Herzinsuffizienz**

Herzinsuffizienz

(ADHS als Exposition: OR = 1,03, 95 %-KI: 1,0–1,04; Herzinsuffizienz als Exposition: OR = 1,2, 95 %-KI: 1,0–1,15; [11]). Ein höherer polygenetischer Risikoscore (PRS) für ADHS war statistisch signifikant mit mehreren kardiometabolischen Erkrankungen (DM Typ II, Adipositas, Bluthochdruck, Thrombosen und Zustand nach koronarem Bypass) und entsprechenden kardiometabolischen Biomarkern (Glukosemetabolismus, Erythrozytenanzahl, Lipidmetabolismus, Leber- und Nierenfunktion, Inflammation und Blutdruck) assoziiert [12].

### Dopaminstoffwechsel

Dopamin spielt eine entscheidende Rolle bei ADHS und der Regulierung von **Appetit und Stoffwechsel**

Appetit und Stoffwechsel.

Es wird angenommen, dass eine Dysregulation des Dopaminsignalwegs sowohl zu ADHS-Symptomen als auch zu einem erhöhten Risiko für Adipositas und andere metabolische Störungen beiträgt (für ein Review siehe [6]).

### Störungen des zirkadianen Rhythmus

Schlafstörungen sind sowohl mit ADHS als auch mit metabolischen Störungen verbunden. Alterierte zirkadiane Funktionen können zu Veränderungen der Essgewohnheiten und zu Veränderungen der **Stoffwechselhormone**

Stoffwechselhormone

beitragen [13, 14].

### Inflammatorische Prozesse

Es gibt zunehmende Hinweise für die Beteiligung von Entzündungsprozessen sowohl bei ADHS (zumindest in Subgruppen; [15]) als auch bei metabolischen Störungen [16]. Erhöhte Spiegel **proinflammatorischer Zytokine**

proinflammatorischer Zytokine

wurden bei ADHS-Patienten [17] und bei Patienten mit metabolischen Störungen [18] beobachtet.

### Lebensstilfaktoren

ADHS-Patienten haben häufig einen **ungesunden Lebensstil**

ungesunden Lebensstil,

darunter ungesunde Ernährung, Bewegungsmangel und Substanzmissbrauch. Diese Lebensstilfaktoren können zum erhöhten Risiko für kardiometabolische Störungen beitragen. Kinder mit ADHS scheinen z. B. oft weniger Nahrungsmittel mit mehrfach ungesättigten Fettsäuren („polyunsaturated fatty acids“, PUFAs) wie **ω‑3-Fettsäuren**

ω‑3-Fettsäuren

zu sich nehmen als Kinder ohne ADHS. Ältere Untersuchungen fanden bei Kindern mit ADHS ein signifikant erhöhtes ω‑6-zu-ω-3-Verhältnis im Serum. Dies korrelierte mit einer erhöhten Aufnahme von nährstoffarmen, zucker- und **fettreichen Lebensmitteln**

fettreichen Lebensmitteln,

was dann auch wieder zu einem erhöhten Risiko für kardiometabolische Erkrankungen später im Leben führen kann [19, 20].

Eine weitere Studie fand, dass ADHS-Kinder im Vergleich zur Kontrollgruppe deutlich mehr **Einfachzucker**

Einfachzucker,

Tee und Fertiggerichte, aber weniger Eiweiß, Vitamin B1, Vitamin B2, Vitamin C, Zink und Kalzium konsumierten [21]. In einer Kohorte von über 700 Jugendlichen mit ADHS konnte gezeigt werden, dass diese im Vergleich zu ihren Peers ohne ADHS-Diagnose signifikant häufiger **Raucher**

Raucher

waren (OR 2,15; 95 %-KI: 1,65–2,80), häufiger bzw. mehr **Alkohol**

Alkohol

konsumierten (OR 1,38; 95 %-KI: 1,10–1,73) und eine exzessivere **Bildschirmnutzung**

Bildschirmnutzung

aufwiesen (OR 1,41; 95 %-KI: 1,18–1,70). Ein ungesunder Lebensstil war in der ADHS-Gruppe generell doppelt so häufig im Vergleich zu den gesunden Kontrollen anzutreffen (OR 2,03; 95 %-KI: 1,64–2,51; [22]).

Es erscheint zunächst auch naheliegend, dass die impulsive und unaufmerksame Symptomatik zu Adipositas bei ADHS beiträgt. Eine beeinträchtigte **inhibitorische Kontrolle**

inhibitorische Kontrolle

(aufgrund von Impulstendenzen) könnte zu übermäßigem Essen, nächtlichem Essen, Naschen und einer Vorliebe für Fastfood sowie kohlenhydratreiche Lebensmittel (d. h. hochverarbeitete Lebensmittel und fett- und zuckerreiche Mahlzeiten) führen. Eine verminderte Aufmerksamkeit könnte zu einem mangelnden Bewusstsein für die Nahrungsaufnahme und einer **schlechten Essensplanung**

schlechten Essensplanung

führen, was zu unregelmäßigen Essgewohnheiten beiträgt (für ein Review siehe [6]). Eine aktuelle Studie, bei der fast 3000 Teilnehmer von der Kindheit bis ins Erwachsenenalter begleitet wurden, fand allerdings keine Belege für einen direkten Zusammenhang zwischen Aufmerksamkeitsproblemen und Hyperaktivität/Impulsivität und späterer Adipositas oder umgekehrt. Diese Ergebnisse deuten entweder auf einen Kausalpfad von ADHS zu Adipositas und/oder umgekehrt hin, der bereits in der frühen Kindheit etabliert wurde, oder auf einen gemeinsamen genetischen Hintergrund [23], wie oben schon erwähnt wurde.

#### Cave

Eine Aufklärung über regemäßige moderate körperliche Aktivität (z. B. nach den WHO[World Health Organization]-Empfehlungen), eine ausgeglichene Ernährung (z. B. Mittelmeer-Diät orientiert, Vermeidung hochprozessierter Lebensmittel) und Rauchverzicht kann hilfreich sein, insbesondere da ADHS-Betroffene ein genetisch erhöhtes Risiko für kardiometabolische Erkrankungen haben und darüber aufgeklärt werden sollten. Noch gibt es keine ausreichend gute Datenlage, um speziellere Ernährungsformen zu empfehlen.

## Behandlung

### Kardiometabolische Nebenwirkungen von ADHS-Medikamenten

**Stimulanzien**

Stimulanzien

können bei ADHS-Patienten zu **Gewichtsverlust**

Gewichtsverlust

führen und die Häufigkeit von Adipositas normalisieren [24]. Eine Metaanalyse zeigte, dass ADHS bei pharmakologisch behandelten Patienten nicht mehr signifikant mit Adipositas in Verbindung gebracht wurde [25]. In einer älteren Tierstudie wurde allerdings gezeigt, dass **Methylphenidat**

Methylphenidat

die Glukoseaufnahme bei Ratten beeinflussen kann, was einen möglichen Mechanismus für diese Beobachtungen darstellt [26]. Ein solcher Effekt könnte möglicherweise Diabetes-mellitus-induzierte Blutzuckerveränderungen verschleiern und eine rechtzeitige Intervention verhindern. Ob dies bei Menschen aber von klinischer Relevanz ist, ist eher fragwürdig, entsprechende Studien fehlen aber.

Stimulanzien und **Atomoxetin**

Atomoxetin

können bei Kindern, Jugendlichen und Erwachsenen zu einem **Anstieg des Blutdrucks**

Anstieg des Blutdrucks

und der Herzfrequenz führen [27]. Eine Metaanalyse zeigte, dass die Behandlung von ADHS mit Stimulanzien zu einem statistisch signifikanten Anstieg der **Herzfrequenz**

Herzfrequenz

(um durchschnittlich 5,7 Schläge pro Minute) und des systolischen Blutdrucks (um durchschnittlich 2,0 mm Hg) führt [28]. Bei Patienten, die mit Stimulanzien behandelt wurden, war das Risiko für eine Ruheherzfrequenz von über 90 Schlägen pro Minute signifikant erhöht (OR 2,75; [28]). Bei der Mehrheit der Patienten sind diese Erhöhungen jedoch nicht von klinischer Bedeutung, und es gibt keine Hinweise auf eine erhöhte kardiovaskuläre Mortalität aufgrund von ADHS-Medikamenten (für ein Review siehe [3]). In einer neueren systematischen Übersichtsarbeit wurde ebenfalls berichtet, dass ADHS-Medikamente mit einer moderaten Erhöhung der Ruheherzfrequenz und des Blutdrucks in Verbindung gebracht werden. Es wurden unerwünschte Wirkungen im Zusammenhang mit Stimulanzien veröffentlicht, darunter Arrhythmien, nichtischämische Kardiomyopathie, Tako-Tsubo-Kardiomyopathie und plötzlicher Tod. Die Autoren kamen jedoch zu dem Schluss, dass es keine überzeugenden Beweise für einen kausalen Mechanismus gibt [29].

**Guanfacin**

Guanfacin

führt aufgrund seiner Eigenschaft als zentral wirksamer Alpha-2- und TAAR1-Rezeptoragonist eher zu einer Abnahme der Herzfrequenz (**Bradykardie**

Bradykardie).

Es kann zu Hypotonie und orthostatischer Hypotonie kommen. Mögliche QTc-Intervall-Verlängerung, aber von unklarer klinischer Relevanz wurden beschrieben. Nach Absetzen kann es zu einem Anstieg von Blutdruck und Pulsfrequenz kommen [30].

#### Merke

Alle Medikamente erfordern eine sorgfältige Überwachung der kardiovaskulären Parameter, besonders zu Behandlungsbeginn und bei Dosisänderungen, insbesondere Blutdruck und Puls.

### Medikamentöse Behandlung von ADHS bei bestehenden kardiometabolischen Erkrankungen

Die Behandlung komorbider ADHS und metabolischer Störungen erfordert einen umfassenden und individualisierten Ansatz.

**Lebensstilinterventionen**

Lebensstilinterventionen

einschließlich Ernährungsberatung, regelmäßiger Bewegung und Schlafhygiene sind sowohl für die Behandlung von ADHS als auch für metabolische Störungen unerlässlich. **Psychotherapie**

Psychotherapie,

wie z. B. kognitive Verhaltenstherapie (KVT), könnte ADHS-Patienten helfen, ungesunde Verhaltensweisen zu ändern, ihre Selbstregulierung zu verbessern und Stress zu bewältigen, wobei hier noch Studien zur Effektivität in diesem speziellen Bereich fehlen.

In Bezug auf die Behandlung eines komorbiden **Diabetes mellitus**

Diabetes mellitus

wurden bisher keine signifikanten Wechselwirkungen zwischen Antidiabetika und Stimulanzien berichtet [31]. Darüber hinaus gibt es Studien, die zeigen, dass komorbide ADHS bei Jugendlichen mit Diabetes mellitus Typ I zu einer schlechteren Blutzuckerkontrolle sowie bei Erwachsenen zu einer schlechteren Adhärenz bei der Insulinpumpentherapie führt [32, 33]. Daher könnte eine suffiziente Behandlung der ADHS auch wichtig für eine bessere Prognose bei somatischen Begleiterkrankungen zu sein.

Bei Kindern mit **angeborenen Herzfehlern**

angeborenen Herzfehlern

und ADHS-Symptomen sind Daten rar und eine Stimulanzienbehandlung muss im Rahmen einer individuellen Risiko-Nutzen-Bewertung in Betracht gezogen werden [34].

Guanfacin hat einen **blutdrucksenkenden Effekt**

blutdrucksenkenden Effekt,

ist aber in Deutschland nur für die Behandlung von Kindern und Jugendlichen zugelassen [35]. Im Einzelfall kann aber eine **Off-label-Behandlung**

Off-label- Behandlung

auch bei ADHS-Erwachsenen mit einem arteriellen Hypertonus eine Option sein, darüber muss aber aufgeklärt werden und es empfiehlt sich einen Antrag auf Kostenübernahme bei der entsprechenden Krankenkasse zu stellen.

#### Merke

Auch Patienten mit vorbestehenden kardiometabolischen Erkrankungen können mit ADHS-Medikamenten behandelt werden, aber es ist besondere Vorsicht geboten und es sollte eine engmaschige Abstimmung mit dem Kardiologen oder Internisten erfolgen. Zudem sollte eine spezifische Aufklärung der Patienten über die potenziellen Risiken erfolgen.

## Fazit für die Praxis

- Menschen mit einer Aufmerksamkeitsdefizit‑/Hyperaktivitätsstörung (ADHS) haben über die Lebensspanne ein erhöhtes Risiko für Adipositas, Diabetes mellitus und kardiovaskuläre Erkrankungen.
- Die Ursache des erhöhten Risikos ist komplex und eine Kombination aus genetischen und Lebensstilfaktoren.
- Stimulanzien können dabei helfen, Gewicht zu reduzieren und eine gesündere Lebensweise einzuhalten.
- Der Einsatz von Stimulanzien ist auch bei komorbiden kardiovaskulären Erkrankungen möglich, allerdings nur nach sorgfältiger Patientenaufklärung und unter enger Absprache mit dem Kardiologen.
- Generell sollte zum einen zur Prävention, aber auch zur Behandlung bereits bestehender komorbider kardiometabolischer Erkrankungen ein individualisiertes multimodales Therapieangebot bereitgestellt werden.

## CME-Fragebogen

Welche der folgenden somatischen Erkrankungen tritt bei ADHS(Aufmerksamkeitsdefizit‐/Hyperaktivitätsstörung)-Patienten eher *nicht* häufiger auf?

Adipositas

Diabetes mellitus Typ II

Arterielle Hypertonie

Triglyzerid-Erhöhung

Chronisch-myeloische Leukämie

Welcher der folgenden Faktoren wird *nicht* als möglicher Pathomechanismus für den Zusammenhang zwischen der Aufmerksamkeitsdefizit‐/Hyperaktivitätsstörung (ADHS) und metabolischen Störungen angesehen?

Gemeinsame Risikogene

Dopaminstoffwechselstörungen

Störungen des zirkadianen Rhythmus

Erhöhte sportliche Aktivität

Inflammatorische Prozesse

Um wie viel Prozent ist die gepoolte Prävalenz von Adipositas bei ADHS(Aufmerksamkeitsdefizit‑/Hyperaktivitätsstörung)-Patienten erhöht (Kinder und Jugendliche)?

10 %

20 %

40 %

60 %

80 %

Welche Aussage zu Stimulanzien und kardiometabolischen Risiken ist richtig?

Stimulanzien führen immer zu einer Gewichtszunahme.

Stimulanzien sind bei Patienten mit bestehenden Herzerkrankungen absolut kontraindiziert.

Stimulanzien können bei einigen Patienten zu einem leichten Anstieg von Herzfrequenz und Blutdruck führen.

Stimulanzien haben keinen Einfluss auf den Glukosestoffwechsel.

Stimulanzien senken immer den Blutdruck.

Welche der folgenden Empfehlungen ist für ADHS(Aufmerksamkeitsdefizit‑/Hyperaktivitätsstörung)-Patienten mit komorbiden kardiometabolischen Störungen *am wenigsten* geeignet?

Lebensstilinterventionen (Ernährung, Bewegung)

Psychotherapie (z. B. kognitive Verhaltenstherapie [KVT])

Regelmäßige Überwachung der kardiovaskulären Parameter

Monotherapie mit hoch dosierten Stimulanzien ohne weitere Maßnahmen

Enge Abstimmung mit Kardiologen oder Internisten

Welche Aussage zu kardiovaskulären Erkrankungen bei einer Aufmerksamkeitsdefizit‐/Hyperaktivitätsstörung (ADHS) trifft *nicht* zu?

ADHS-Betroffene haben ein erhöhtes Risiko für arterielle Hypertonie.

ADHS-Betroffene haben ein erhöhtes Risiko für Myokardinfarkt.

ADHS-Betroffene haben ein erhöhtes Risiko für Herzinsuffizienz.

ADHS-Betroffene haben ein erhöhtes Risiko für Herzmyxome.

ADHS-Betroffene haben ein erhöhtes Risiko für Vorhofflimmern.

Welche Substanz wird aufgrund seiner Eigenschaft als zentral wirksamer Alpha-2- und TAAR1-Rezeptoragonist eher zu einer Abnahme der Herzfrequenz führen?

Methylphenidat

Atomoxetin

Lisdexamfetamin

Guanfacin

Koffein

Welche Aussage zu genetischen Faktoren bei einer Aufmerksamkeitsdefizit‑/Hyperaktivitätsstörung (ADHS) und metabolischen Erkrankungen ist korrekt?

Es gibt keine genetischen Verbindungen zwischen ADHS und metabolischen Erkrankungen.

Genomweite Assoziationsstudien (GWAS) haben keine genetischen Korrelationen zwischen ADHS und dem Body-Mass-Index (BMI) gefunden.

Ein höherer polygenetischer Risikoscore (PRS) für ADHS ist statistisch signifikant mit mehreren kardiometabolischen Erkrankungen assoziiert.

Genetische Faktoren spielen nur bei Kindern, nicht aber bei Erwachsenen eine Rolle.

ADHS und metabolische Störungen haben komplett unterschiedliche genetische Ursachen.

Welchen Effekt haben Stimulanzien in Bezug auf Gewichtsverlust?

Stimulanzien führen meistens zu einer Gewichtszunahme.

Stimulanzien können zu Gewichtsverlust führen.

Stimulanzien haben in der Regel keinen Einfluss auf das Gewicht.

Nur Atomoxetin führt zu Gewichtsverlust.

Nur Lisdexamfetamin führt zu Gewichtsverlust.

Ein 25-jähriger Patient mit ADHS wird mit 40 mg Methylphenidat retard behandelt. Er berichtet darunter von einer gewünschten Appetitzügelung, aber er hätte sich einen größeren Effekt auf die Reduktion seines Übergewichts (aktuell BMI 38) gewünscht. Welche Aussage in der Beratung ist am ehesten zielführend?

Absetzen von Methylphenidat und Umstellung auf Atomoxetin

Kontrollierter Beginn eines mäßigen Nikotinkonsums

Bestimmung von Glucagon-like-Peptide Polymorphismen im Blut

Individualisierter Ansatz mit Empfehlung Psychotherapie, Abklärung Diabetes, Hypertonus, Dyslipidämie. Empfehlung körperliche Aktivität, Ernährung

Behandlung mit Metformin und Umstellung auf Lisdexamphetamin
